# QUALITY IMPROVEMENT IN DEMENTIA CARE: EFFECTIVENESS OF IMPLEMENTING STAR-VA IN A VA COMMUNITY LIVING CENTER

**DOI:** 10.1093/geroni/igz038.3531

**Published:** 2019-11-08

**Authors:** Thomas Chacko, Kim Curyto, Marc Maller, Connie Eddy

**Affiliations:** 1 University at Buffalo, New York, United States; 2 Center for Integrated Healthcare, VA Western New York Healthcare System, Batavia, New York, United States; 3 VA Western NY Healthcare System, New York, United States

## Abstract

Staff Training in Assisted Living Residences adapted for Veterans Affairs (STAR-VA) is an evidence-based, interdisciplinary behavioral intervention to manage challenging Behavioral Symptoms of Dementia (BSD) at VA Community Living Centers (CLCs). Components of STAR-VA included creating realistic expectations for individuals with dementia, effective communication, using a behavioral problem-solving approach, and increasing resident-centered pleasant events. STAR-VA was implemented at VA Western New York CLC to help staff manage Veteran BSD. The purpose of the study was to examine the effectiveness of STAR-VA implementation in 17 CLC Veterans with Dementia who demonstrated BSD distressing to themselves, other Veterans and/or staff and documented in 2017-2018. STAR-VA was facilitated by the behavioral coordinator and nurse champion in partnership with the CLC behavior team during weekly rounds and as needed modeling and debriefing. The team included the medical provider, recreation therapist, social worker, and all levels of nursing. On average, the intervention involved four assessments over 75 days. Outcome measures at baseline and post-intervention included assessments of target behavior frequency and severity, direct care tracking of behaviors, and the Minimum Data Set distress behavior indicator. Compared to baseline scores, clinically meaningful reduction was documented using team assessment and direct care ratings of BSD frequency and severity, and overall inappropriate utilization of antipsychotic medication. CLC clinical staff tailored implementation of STAR-VA to be feasible and effective. Ongoing evaluation of STAR-VA implementation using routine measures promotes measurement based care, providing feedback to the team to improve care quality.

